# The affine Pólya–Szegö principle: Equality cases and stability^☆^

**DOI:** 10.1016/j.jfa.2013.06.001

**Published:** 2013-10-15

**Authors:** Tuo Wang

**Affiliations:** Institut für Diskrete Mathematik und Geometrie, TU Wien, Wiedner Hauptstrasse 8-10, 1040 Wien, Austria

**Keywords:** Affine Pólya–Szegö, Stability, Functional Minkowski problem

## Abstract

A Brothers–Ziemer type theorem for the affine Pólya–Szegö principle and a quantitative affine Pólya–Szegö principle are established.

## 1 Introduction

The classical Pólya–Szegö principle states that the $L^{p}$ norm of the gradient of a function on $R^{n}$ does not increase under Schwarz radially decreasing symmetrization. It plays a fundamental role in the solution to a number of variational problems in different areas such as isoperimetric inequalities, optimal forms of Sobolev inequalities, and sharp a priori estimates of solutions to second-order elliptic or parabolic boundary value problems (see, for example, [36], [37], [38], [54], [55] and the references therein).

A strengthened version of the classical Pólya–Szegö principle has been established by Cianchi, Lutwak, Yang, and Zhang (see, e.g., [16], [47], [49]). The strengthened principle is discovered through the $L^{p}$ Brunn–Minkowski theory and it is remarkable that it is invariant under the action of the special linear group.

To be precise, for $f\in W^{1,p}(R^{n})$, the usual Sobolev spaces, the $L^{p}$ affine energy $\Omega_{p}(f)$ is defined [16], by

$$\Omega_{p}(f)=c_{n,p}{\left(\int_{S^{n-1}}{\left\|D_{u}f\right\|}_{p}^{-n}\;du\right)}^{-1/n},$$

where $c_{n,p}={\left(n\omega_{n}\right)}^{1/n}{\left(\frac{n\omega_{n}\omega_{p-1}}{2\omega_{n+p-2}}\right)}^{1/p}$, $\omega_{n}=\frac{\pi^{n/2}}{\Gamma (1+\frac{n}{2})}$, ${\left\|\cdot \right\|}_{p}$ is the usual $L^{p}$ norm on $R^{n}$ and $D_{u}(f)$ is the directional derivative of *f* defined by

$$D_{u}f=u\cdot \nabla f.$$

Given $f\in W^{1,p}(R^{n})$, its distribution function $\mu_{f}:[0,\infty )\to [0,\infty ]$ is defined by

$$\mu_{f}(t)=\left|\left\{x\in R^{n}:\left|f(x)\right|>t\right\}\right|,$$

where |⋅| denotes Lebesgue measure on $R^{n}$. The decreasing rearrangement $f^{⁎}:[0,\infty )\to [0,\infty ]$ of *f* is defined by

$$f^{⁎}(s)=\inf\left\{t>0:\mu_{f}(t)⩽s\right\}.$$

The symmetric rearrangement of *f* is the function $f^{⋆}:R^{n}\to [0,\infty ]$ defined by

$$f^{⋆}(x)=f^{⁎}\left(\omega_{n}{\left|x\right|}^{n}\right),$$

where |⋅| is the standard Euclidean norm.

For an origin-symmetric convex body *K*, the convex symmetrization $f^{K}$ of *f* with respect to *K* is defined as follows:

$$f^{K}(x)=f^{⁎}\left(\omega_{n}{\left\|x\right\|}_{\tilde{K}}^{n}\right),$$

where ${\left\|\cdot \right\|}_{\tilde{K}}$ is the Minkowski functional of $\tilde{K}$, with $\tilde{K}$ being a dilation of *K* so that $|\tilde{K}|=\omega_{n}$.

The classical Pólya–Szegö principle could be stated as

(1) $${\left\|\nabla f\right\|}_{p}⩾{\left\|\nabla f^{⋆}\right\|}_{p},$$

here ∇*f* is the weak derivative of *f* on $R^{n}$.

And the affine Pólya–Szegö principle established by Cianchi, Lutwak, Yang and Zhang [51] states that if $f\in W^{1,p}(R^{n})$, then

(2) $$\Omega_{p}(f)⩾\Omega_{p}\left(f^{⋆}\right).$$

The affine energy $\Omega_{p}(\cdot )$ is invariant under the action of the special linear group. And using the Hölder inequality, one sees that the affine Pólya–Szegö principle is stronger than the Euclidean counterpart in the sense that

(3) $${\left\|\nabla f\right\|}_{p}⩾\Omega_{p}(f)⩾\Omega_{p}\left(f^{⋆}\right)={\left\|\nabla f^{⋆}\right\|}_{p}.$$

As pointed out in [13], investigations on the uniqueness of the relevant extremals in Pólya–Szegö type principles are more recent, and typically require an additional delicate analysis. Such a description has first been the object of the series of papers [7], [26], [36], [57] and later has been recently extended and simplified by new contributions, including [8], [9], [12], [14], [17], [18], [20], [22], [21]. It is the aim of this paper that we study the uniqueness of extremals in (2).

In the Euclidean case (1), partial results are contained in [36] and [57]. The problem was also discussed by Friedman and Mcleod in [26] when *f* is of class $C^{n}$. However, as observed in [7], the proof in [26] contains an error which can be only repaired under additional assumptions. A general answer was given by Brothers and Ziemer [7], where the following theorem is proved.

Theorem 1 *(See* *[7]* *.) Let f be a compactly supported nonnegative function from* $W^{1,p}(R^{n})$ *,* 1<p<+∞ *, such that*

(4) $$\left|\left\{\left|Df^{⋆}\right|=0\right\}\cap {\left(f^{⋆}\right)}^{-1}(0,\mathrm{ess}\sup f)\right|=0.$$

*If* ${\left\|\nabla f^{⋆}\right\|}_{p}={\left\|\nabla f\right\|}_{p}$ *, then*

$$f=f^{⋆}\;\text{a.e. in}R^{n},$$

*up to a translation.*

In [7], it is also shown that condition (4) is equivalent to the absolute continuity of $\mu_{f}$. And Brothers and Ziemer also constructed a smooth functions *f*, where ${\left\|\nabla f\right\|}_{p}={\left\|\nabla f^{⋆}\right\|}_{p}$ without *f* being any translate of $f^{⋆}$, if one does not assume condition (4).

Thus in view of (3), the example constructed by Brothers and Ziemer would satisfy $\Omega_{p}(f)=\Omega_{p}(f^{⋆})$, but *f* fails to be any translate of symmetrization of *f* in any suitable sense. Thus absolute continuity of $\mu_{f}$ is a necessary condition for our investigation of the extremals of (2), and it turns out to be a sufficient condition! In particular we establish the following theorem.

Theorem 2 *If f is a nonnegative function from* $W^{1,p}(R^{n})$ *,* 1<p<+∞ *, such that*

$$\left|\left(\left\{\left|\nabla f^{⋆}\right|=0\right\}\cap \left\{0<f^{⋆}<\mathrm{ess}\sup f\right\}\right)\right|=0,$$

*then*

$$\Omega_{p}(f)=\Omega_{p}\left(f^{⋆}\right)$$

*if and only if there exists* $x_{0}\in R^{n}$ *such that* $f(x)=f^{E}(x+x_{0})$ *a.e. in* $R^{n}$ *, here E is an origin-centered ellipsoid in* $R^{n}$ *.*

The Euclidean Sobolev deficit of a function $f\in W^{1,p}(R^{n})$ is defined as the translation invariant quantity

$$D_{e}(f)=\frac{{\left\|\nabla f\right\|}_{p}}{{\left\|\nabla f^{⋆}\right\|}_{p}}-1.$$

Finding a quantitative version of (1) consists in proving that the “Euclidean Sobolev deficit” controls a suitable notion of “distance from the family of the extremal functions”. The following Euclidean asymmetry of the function *f* is introduced in [13], where it is defined by

$$A_{e}(f)=\min_{\pm }\underset{x_{0}\in R^{n}}{\inf}{\left\|f(\cdot )\pm f^{⋆}(\cdot +x_{0})\right\|}_{1},$$

and ${\left\|\cdot \right\|}_{1}$ denotes the $L^{1}$ difference. Note this asymmetry is also invariant under translations.

It is established in [13] that

$$A_{e}(f)⩽C{\left[M_{f}\left(D_{e}{\left(f\right)}^{r}\right)+D_{e}(f)\right]}^{s},$$

where C,r,s are positive constants depending on *n* and *p*, $M_{f}$ is defined by

$$M_{f}(\sigma )=\frac{\left|(\{|\nabla f|⩽\sigma \}\cap \{0<|f|<\mathrm{ess}\sup|f|\})\right|}{\left|(\{|f|>0\})\right|}$$

for σ⩾0, *f* is normalized such that |{|f|>0}|=1 and ${\left\|\nabla f\right\|}_{p}=1$.

We shall not attempt here to sketch the history and motivation of this problem, but simply refer to [13] by Cianchi, Esposito, Fusco and Trombetti (and the references therein) where this quantitative version of the Pólya–Szegö principle is established.

The main goal of the second part of this paper is to prove a quantitative version of the affine Pólya–Szegö principle, with the assumption $|\{x\in R^{n}:|f(x)|>0\}|<\infty$. To this purpose we introduce the following affine invariant quantity. For a Sobolev function $f\in W^{1,p}(R^{n})$, we define the $L^{p}$ affine ratio as

$$R_{a}(f):=\frac{\Omega_{p}(f)}{\Omega_{p}(f^{⋆})}.$$

Finding a quantitative version of (3) consists in proving that the “affine ratio” controls a more usual notion of “affine distance from the family of the extremal functions”. To this aim is introduced the affine asymmetry of the function *f*, and it is defined by

$$A_{a}(f)=\min_{\pm }\underset{x_{0}\in R^{n},E\in E}{\inf}{\left\|f(\cdot )\pm f^{E}(\cdot +x_{0})\right\|}_{1},$$

where E is the family of all origin-centered ellipsoids. Note that the affine asymmetry is also invariant under affine transformations.

We have the following result:

Theorem 3 *If f is a function in* $W^{1,p}(R^{n})$ *with* |{|f|>0}|=1 *and* ${\left\|f\right\|}_{W^{1,p}(R^{n})}=1$ *, then*

$$A_{a}(f)⩽C{\left[M_{f}(\sigma )+\frac{\sigma +1}{\sigma }{\left(R_{a}{\left(f\right)}^{p}-1\right)}^{r_{1}}\right]}^{r_{2}}+2\left(e^{r_{3}\frac{R_{a}{\left(f\right)}^{n}-1}{R_{a}{\left(f\right)}^{n}}}-1\right),$$

*for every* σ>0 *, where* $C,r_{1},r_{2},r_{3}$ *are positive constants depending only on n and p and* ${\left\|f\right\|}_{W^{1,p}(R^{n})}$ *is the usual Sobolev norm of f in* $W^{1,p}(R^{n})$ *defined by* ${\left\|f\right\|}_{W^{1,p}(R^{n})}={\left\|f\right\|}_{p}+{\left\|\nabla f\right\|}_{p}$ *.*

## 2 Background material

### 2.1 Background on $L^{p}$ Brunn–Minkowski theory

For quick reference we recall in this section some background material from the $L^{p}$ Brunn–Minkowski theory of convex bodies. This theory has its origin in the work of Firey from the 1960ʼs and has expanded rapidly over the last two decades since the work of Lutwak [44] (see, e.g., [10], [11], [31], [32], [35], [39], [40], [41], [42], [44], [45], [46], [51], [47], [49], [48], [50], [58], [59]).

A convex body is a compact convex subset of $R^{n}$ with non-empty interior. We write $K^{n}$ for the set of convex bodies in $R^{n}$ endowed with the Hausdorff metric and we denote by $K_{e}^{n}$ the set of origin-symmetric convex bodies and by $K_{0}^{n}$ the set of convex bodies containing origin in the interior. Each non-empty compact convex set *K* is uniquely determined by its support function $h_{K}(\cdot )$, defined by

$$h_{K}(x)=\max\{x\cdot y:y\in K\},\;x\in R^{n},$$

where x⋅y denotes the usual inner product of *x* and *y* in $R^{n}$. Note that $h_{K}(\cdot )$ is positively homogeneous of degree one and sub-additive. Conversely, every function with these properties is the support function of a unique compact convex set.

The Banach–Mazur distance $\delta_{BM}(K,L)$ of the convex bodies *K* and *L* is defined by

$$\delta_{BM}(K,L)=\min\left\{\lambda ⩾0:K-x\subset \psi (L-y)\subset e^{\lambda }(K-x)\text{ for some }\psi \in GL(n),\;x,y\in R^{n}\right\}.$$

In particular, if *K* and *L* are origin-symmetric, then x=y=0 can be assumed.

If $K\in K_{0}^{n}$, then the polar body $K^{\circ }$ of *K* is defined by

$$K^{\circ }=\left\{x\in R^{n}:x\cdot y⩽1\text{ for all }y\in K\right\}.$$

From the polar formula for volume it follows that the *n*-dimensional Lebesgue measure $\left|K^{\circ }\right|$ of the polar body $K^{\circ }$ can be computed by

$$\left|K^{\circ }\right|=\frac{1}{n}\int_{S^{n-1}}h_{K}{\left(u\right)}^{-n}\;du,$$

where integration is with respect to spherical Lebesgue measure.

For real p⩾1 and α,β>0, the $L^{p}$ Minkowski combination of $K,L\in K_{0}^{n}$ is the convex body $\alpha \cdot K{+}_{p}\beta \cdot L$ defined by

$$h_{\alpha \cdot K{+}_{p}\beta \cdot L}{\left(\cdot \right)}^{p}=\alpha h_{K}{\left(\cdot \right)}^{p}+\beta h_{L}{\left(\cdot \right)}^{p}.$$

The $L^{p}$ mixed volume $V_{p}(K,L)$ of $K,L\in K_{0}^{n}$ was defined in [44] by

$$V_{p}(K,L)=\frac{p}{n}\lim_{\epsilon \to 0^{+}}\frac{\left|K{+}_{p}\epsilon \cdot L|-|K\right|}{\epsilon }.$$

Clearly, the diagonal form of $V_{p}$ reduces to ordinary volume, i.e., for $K\in K_{0}^{n}$,

$$V_{p}(K,K)=|K|.$$

It was also shown in [44] that for all convex bodies $K,L\in K_{0}^{n}$,

$$V_{p}(K,L)=\frac{1}{n}\int_{S^{n-1}}h_{L}{\left(u\right)}^{p}\;dS_{p}(K,u),$$

where $dS_{p}(K,u)=h_{K}{\left(u\right)}^{1-p}\;dS(K,u)$ and the measure S(K,⋅) on $S^{n-1}$ is the classical surface area measure of *K*. Recall that for a Borel set $\omega \subset S^{n-1}$, S(K,ω) is the (n−1)-dimensional Hausdorff measure of the set of all boundary points of *K* for which there exists a normal vector of *K* belonging to *ω*.

Projection bodies have become a central notion within the Brunn–Minkowski theory. They arise naturally in a number of different areas such as functional analysis, stochastic geometry and geometric tomography. The fundamental affine isoperimetric inequality which connects the volume of a convex body with that of its polar projection body is the Petty projection inequality [53]. This inequality turned out to be far stronger than the classical isoperimetric inequality and has led to Zhangʼs affine Sobolev inequality [61].

The $L^{p}$ projection body $\Pi_{p}K$ of $K\in K_{0}^{n}$, was introduced in [45] as the convex body such that

$$h_{\Pi_{p}K}{\left(u\right)}^{p}=\int_{S^{n-1}}{\left|u\cdot v\right|}^{p}\;dS_{p}(K,v),\;u\in S^{n-1}.$$

The $L^{p}$ analog of Pettyʼs projection inequality plays a key role in the $L^{p}$ Brunn–Minkowski theory. It was first proved by Lutwak, Yang, and Zhang [47] (see also Campi and Gronchi [10] for an independent approach): If $K\in K_{0}^{n}$, then

$${\left|K\right|}^{n/p-1}\left|\Pi_{p}^{\circ }K\right|⩽{\left|B_{1}\right|}^{n/p-1}\left|\Pi_{p}^{\circ }B_{1}\right|,$$

where $B_{1}$ is the unit ball and equality is attained if and only if *K* is an ellipsoid centered at the origin.

The classical Minkowski problem also has a counterpart in the $L^{p}$ Brunn–Minkowski theory. The even $L^{p}$ Minkowski problem asks the following: Given an even Borel measure *μ* on $S^{n-1}$, does there exist a convex body *K* such that $\mu =S_{p}(K,\cdot )$?

Lutwak [44] gave an affirmative answer to this problem when p≠n. The authors [50] introduced the volume-normalized $L^{p}$ Minkowski problem, for which the case p=n can be handled as well. In particular, the following is proved:

Theorem 4 *(See* *[50]* *.) If* 1⩽p<∞ *and μ is an even Borel measure on the unit sphere* $S^{n-1}$ *, then there exists a unique origin-symmetric convex body K such that*

$$\frac{S_{p}(K,\cdot )}{\left|K\right|}=\mu$$

*if and only if the support of μ is not contained in any* (n−1) *-dimensional linear subspace.*

### 2.2 Anisotropic perimeter

We briefly recall that a Lebesgue set *E* of finite Lebesgue measure is said to be of finite perimeter if its characteristic function $\chi_{E}$ belongs to $BV(R^{n})$, and then the Euclidean perimeter P(E) of *E* is given by the total variation of the distributional derivative of $\chi_{E}$. Throughout this paper, we shall refer to the monograph [2] for the basic properties of sets of finite perimeter.

The anisotropic isoperimetric inequality arises in connection with a natural generalization of the Euclidean notion of perimeter. If *E* is a set of finite perimeter in $R^{n}$, then its anisotropic perimeter, with respect to a convex body $K\in K_{0}^{n}$, is defined as

$$P_{K}(E)=\int_{\partial^{⋆}E}h_{K}\left(\nu_{E}(x)\right)\;dH^{n-1}(x),$$

where $H^{n-1}$ is the (n−1)-dimensional Hausdorff measure, $\partial^{⋆}E$ denotes the reduced boundary of *E* and $\nu_{E}:\partial^{⋆}E\to S^{n-1}$ is the measure-theoretic outer unit normal vector field to *E* (see [2] for more details).

The anisotropic isoperimetric inequality states that,

(5) $$P_{K}(E)⩾n{\left|K\right|}^{1/n}{\left|E\right|}^{1-1/n},$$

for any $E\subset R^{n}$ of sets of finite perimeter.

It was conjectured by Wulff [60] in 1901, that the equality holds if and only if *E* is homothetic to *K*. And it was first shown that this is indeed the case by Taylor [56], and later, with an alternative proof, by Fonseca and Müller [25]. Very recently, Figalli, Maggi and Pratelli [24] obtained an optimal quantitative version of this inequality.

## 3 Extremal cases

As pointed out in [21], even if the method of Brothers and Ziemer is based on a geometrically clear approach, the rigorous justification of the arguments of [7] is accomplished after overcoming serious technical difficulties by means of results from geometric measure theory.

The original proof of the affine Pólya–Szegö principle in [16] is based on the $L^{p}$ projection inequality applied to level sets of the Sobolev function. However, unlike the isoperimetric inequality, this inequality is invariant under all special linear transformations. This makes the Brothers–Ziemer type level set analysis much more complicated, since the method by Brothers–Ziemer depends ultimately on the homothety of the extremals in (5)!

We overcome this difficulty by using an approach by Lutwak, Yang and Zhang in [51], in particular, the concept of the functional Minkowski problem on $W^{1,p}(R^{n})$. This will be the starting point of the present work.

### 3.1 $L^{1}$ case

As observed by Brothers and Ziemer [7], in fact, any Lipschitzian function *f* whose level sets are (n−1)-spheres satisfies $\int_{R^{n}}|\nabla f^{⋆}(x)|\;dx=\int_{R^{n}}|\nabla f(x)|\;dx$. For our investigation, we introduce some facts that we will need.

Theorem 5 **Functional Minkowski problem on $W^{1,1}(R^{n})$**

*(See**[51]**.) Given a function*$f\in W^{1,1}(R^{n})$*, there exists a unique origin-symmetric convex body*〈f〉*that*

$$\int_{R^{n}}\Phi \left(-\nabla f(x)\right)\;dx=\int_{S^{n-1}}\Phi (u)\;dS\left(\langle f\rangle ,u\right),$$

*for every continuous function*$\Phi :R^{n}\to [0,\infty )$*that is homogeneous of degree* 1*.*

Theorem 6 **Co-area formula**

*If* $f\in W^{1,1}(R^{n})$ *and* $g:R^{n}\to [0,+\infty ]$ *is a Borel function, then*

$$\int_{R^{n}}g(x)\left|\nabla f(x)\right|\;dx=\int_{-\infty }^{+\infty }\int_{\left\{f=t\right\}}g(x)\;dH^{n-1}(x)\;dt.$$

Theorem 7 *(See* [30], [43], [53] *.) If K is a convex body, then*

$${\left|K\right|}^{n-1}\left|\Pi_{1}^{\circ }K\right|⩽{\left|B_{1}\right|}^{n-1}\left|\Pi_{1}^{\circ }B_{1}\right|$$

*with equality if and only if K is an ellipsoid. Here* $\Pi_{1}^{\circ }K$ *stands for the polar projection body of K and* $B_{1}$ *is the unit ball of* $R^{n}$ *.*

Lemma 1 *(See* *[7]* *.) For a nonnegative* $f\in W^{1,1}(R^{n})$ *,* {x:f(x)=t} *is the reduced boundary of* {x:f(x)>t} *up to an* $H^{n-1}$ *-negligible set for almost all* t∈[0,∞) *.*

Lemma 2 *(See* *[19]* *.) For positive* $f\in W^{1,1}(R^{n})$ *and almost all* t∈[0,∞) *,* $\frac{-\nabla f(x)}{\left|\nabla f(x)\right|}$ *is the measure-theoretic outer normal to* {x:f(x)>t} *for* {x:f(x)=t} *up to an* $H^{n-1}$ *-negligible set.*

Theorem 8 *For nonnegative* $f\in W^{1,1}(R^{n})$ *, we have* $\Omega_{1}(f)=\Omega_{1}(f^{⋆})$ *if and only if the level sets* ${\left[f\right]}_{t}=\{x:f(x)⩾t\}$ *are homothetic ellipsoids up to an* $L^{n}$ *-negligible set for almost all* t∈[0,∞) *.*

Proof By Theorem 5, we have

$$\int_{S^{n-1}}\Phi (u)\;dS\left(\langle f\rangle ,u\right)=\int_{R^{n}}\Phi \left(\nabla f(x)\right)\;dx$$

for any 1-homogeneous continuous function $\Phi :R^{n}\to [0,\infty )$.

By the co-area formula, we have

$$\int_{R^{n}}\Phi \left(\nabla f(x)\right)\;dx=\int_{-\infty }^{+\infty }\int_{f=t}\Phi \left(\frac{\nabla f(x)}{\left|\nabla f(x)\right|}\right)\;dH^{n-1}\;dt.$$

By taking $\Phi =h_{\langle f\rangle }$, recalling Lemma 1, Lemma 2 and (5), we get

$$n\left|\langle f\rangle \right|=\int_{S^{n-1}}h_{\langle f\rangle }(x)\;dS\left(\langle f\rangle ,x\right)=\int_{0}^{+\infty }P_{\langle f\rangle }\left({\left[f\right]}_{t}\right)\;dt⩾n\int_{0}^{+\infty }{\left|\langle f\rangle \right|}^{1/n}{\left|{\left[f\right]}_{t}\right|}^{1/n^{\prime }}\;dt.$$

Therefore, ${\left|\langle f\rangle \right|}^{1/n^{\prime }}⩾\int_{0}^{+\infty }{\left|{\left[f\right]}_{t}\right|}^{1/n^{\prime }}\;dt$ and there is equality if and only if ${\left[f\right]}_{t}$ is homothetic to 〈f〉 for almost every t∈R.

Therefore, by Theorem 7, we have

(6) $$C_{n}{\left|\Pi^{\circ }\langle f\rangle \right|}^{-1/n}⩾\int_{0}^{+\infty }{\left|{\left[f\right]}_{t}\right|}^{1/n^{\prime }}\;dt,$$

with equality if and only if ${\left[f\right]}_{t}$ is homothetic to an ellipsoid for almost every t∈[0,∞), here $n^{\prime }=\frac{n}{n-1}$, $C_{n}$ is some optimal dimensional constant. We conclude the proof by noting that $A_{1}(f)$ is the product of left hand side of (6) and a dimensional constant.  □

But notice that these ellipsoids need not to be concentric.

### 3.2 $L^{p}$ case for p>1

Theorem 9 **Functional Minkowski problem on $W^{1,p}(R^{n})$**

*(See**[51]**.) Given*1<p<∞*and a function*$f\in W^{1,p}(R^{n})$*, there exists a unique origin-symmetric convex body*${\langle f\rangle }_{p}$*that*

$$\int_{R^{n}}\Phi^{p}\left(-\nabla f(x)\right)\;dx=\frac{1}{\left|{\langle f\rangle }_{p}\right|}\int_{S^{n-1}}\Phi {\left(u\right)}^{p}\;dS_{p}\left({\langle f\rangle }_{p},u\right),$$

*for every continuous function*$\Phi :R^{n}\to [0,\infty )$*that is homogeneous of degree* 1*.*

Following the definition of Lutwak, Yang and Zhang [51], we define the normalized $L^{p}$ projection body ${\tilde{\Pi }}_{p}K$ of *K* by:

$$h_{{\tilde{\Pi }}_{p}K}^{p}(v)=\frac{1}{\left|K\right|}\int_{S^{n-1}}{\left|u\cdot v\right|}^{p}\;dS_{p}(K,u).$$

We observe that $\Omega_{p}(f)=c_{n,p}n^{-\frac{1}{n}}(|{\tilde{\Pi }}_{p}^{\circ }{\langle f\rangle }_{p}{\left|\right)}^{-\frac{1}{n}}$.

The $L^{p}$ Minkowski inequality reads as:

Theorem 10 *(See Lutwak**[44]**.) If*1<p<∞*and*K,L*are origin-symmetric convex bodies in*$R^{n}$*, then*

$$V_{p}(K,L)⩾{\left|K\right|}^{1-p/n}{\left|L\right|}^{p/n}.$$

*And equality holds if and only if*L=tK*for some*t>0*.*

Convex symmetrization behaves well for the functional Minkowski problem:

Lemma 3 *If*$f\in W^{1,p}(R^{n})$*and*K,L*are origin-symmetric convex bodies, then*${\langle f^{K}\rangle }_{p}$*is a dilate of K and*

$$\left|{\langle f^{K}\rangle }_{p}\right|=\left|{\langle f^{L}\rangle }_{p}\right|.$$

Proof Since $h_{K^{\circ }}(\cdot )$ is a Lipschitzian function and $h_{K^{\circ }}(\cdot )=1$ on ∂*K*, for almost all x∈∂K,

$$\sigma_{K}(x)=\frac{\nabla h_{K^{\circ }}(x)}{\left|\nabla h_{K^{\circ }}(x)\right|},$$

where $\sigma_{K}(x)$ is the outer unit normal vector of *K* at the point *x*. And from the definition of the polar body, we have

$$h_{K}\left(\sigma_{K}(x)\right)=\frac{1}{\left|\nabla h_{K^{\circ }}(x)\right|}.$$

Also, it is well known (see [7]) that $f^{⁎}$ is locally absolutely continuous on (0,∞). If *K* is normalized such that $\left|K|=|B_{1}\right|$, we have, by the co-area formula applied to $h_{K^{\circ }}(\cdot )$, that:

$$\int_{R^{n}}\Phi^{p}\left(-\nabla f^{K}(x)\right)\;dx=\int_{R^{n}}\Phi^{p}\left({\left(f^{⁎}\right)}^{\prime }\left(\omega_{n}h_{K^{\circ }}{\left(x\right)}^{n}\right)n\omega_{n}h_{K^{\circ }}{\left(x\right)}^{n-1}\nabla h_{K^{\circ }}(x)\right)\;dx=\int_{0}^{\infty }\int_{\partial K}t^{n-1}{\left(\left(-{\left(f^{⁎}\right)}^{\prime }\left(\omega_{n}t^{n}\right)\right)n\omega_{n}t^{n-1}\right)}^{p}\Phi^{p}\left(\frac{\nabla h_{K^{\circ }}(x)}{\left|\nabla h_{K^{\circ }}(x)\right|}\right){\left|\nabla h_{K^{\circ }}(x)\right|}^{p-1}\;dH^{n-1}(x)\;dt={\left(n\omega_{n}\right)}^{p}\int_{0}^{\infty }t^{np+n-p-1}{\left({\left(-f^{⁎}\right)}^{\prime }\left(\omega_{n}t^{n}\right)\right)}^{p}\;dt\int_{S^{n-1}}\Phi^{p}(u)h^{1-p}(K,u)\;dS(K,u)={\left(n\omega_{n}\right)}^{p}\int_{0}^{\infty }t^{np+n-p-1}{\left({\left(-f^{⁎}\right)}^{\prime }\left(\omega_{n}t^{n}\right)\right)}^{p}\;dt\int_{S^{n-1}}\Phi^{p}(u)\;dS_{p}(K,u)$$

for any *Φ* that is homogeneous of degree 1.

So we get

$$\frac{1}{\left|{\langle f^{K}\rangle }_{p}\right|}S_{p}\left({\langle f^{K}\rangle }_{p},u\right)=C(f)S_{p}(K,u),$$

where $C(f)={\left(n\omega_{n}\right)}^{p}\int_{0}^{\infty }t^{np+n-p-1}{\left({\left(-f^{⁎}\right)}^{\prime }(\omega_{n}t^{n})\right)}^{p}\;dt$.

By comparing the homogeneity degrees of $S_{p}$ and *V*, we get

$${\left|{\langle f^{K}\rangle }_{p}\right|}^{\frac{-p}{n-p}}=C{\left(f\right)}^{\frac{n}{n-p}}|K|.$$

Since $f^{cK}=f^{K}$ for any c∈(0,∞) and any $K\in K_{0}^{n}$, we have

$${\left|{\langle f^{K}\rangle }_{p}\right|}^{\frac{-p}{n-p}}={\left|{\langle f^{L}\rangle }_{p}\right|}^{\frac{-p}{n-p}}.\;□$$

A norm is uniquely determined by its unit ball, which by definition must be an origin-symmetric convex body. Thus we identify a norm with the support function of an origin-symmetric convex body for convenience.

The Pólya–Szegö principle for general Minkowski functionals together with its extremal cases have been established.

Theorem 11 *(See*[1], [20], [22]*.) Let*$K\in K_{0}^{n}$*. If f is a nonnegative function from*$W^{1,p}(R^{n})$*,*1<p<∞*, such that*

$$\left|\left(\left\{\left|\nabla f^{⋆}\right|=0\right\}\cap \left\{0<f^{⋆}<\mathrm{ess}\sup f\right\}\right)\right|=0,$$

*then*

$$\int_{R^{n}}h_{K}^{p}\left(\nabla f(x)\right)\;dx⩾\int_{R^{n}}h_{K}^{p}\left(\nabla f^{K}(x)\right)\;dx,$$

*and*

$$\int_{R^{n}}h_{K}^{p}\left(\nabla f(x)\right)\;dx=\int_{R^{n}}h_{K}^{p}\left(\nabla f^{K}(x)\right)\;dx,$$

*if and only if*$f=f^{K}$*a.e. in*$R^{n}$ (*up to translations*)*.*

Lemma 4 *For*$f\in W^{1,p}(R^{n})$*nonnegative, we have*

$${\langle f\rangle }_{p}\subseteq {\langle f^{{\langle f\rangle }_{p}}\rangle }_{p},$$

*with*${\langle f\rangle }_{p}={\langle f^{{\langle f\rangle }_{p}}\rangle }_{p}$*if and only if*$f=f^{K}$*a.e. in*$R^{n}$ (*up to translations*) *for some* $K\in K_{e}^{n}$*.*

Proof By Theorem 9, we get, for some $K\in K_{e}^{n}$, that

$$\int_{R^{n}}h_{K}^{p}(\nabla f)\;dx=\frac{n}{\left|{\langle f\rangle }_{p}\right|}\left|{\langle f\rangle }_{p},K\right|.$$

By Theorem 11, we have

$$\frac{n}{\left|{\langle f\rangle }_{p}\right|}V_{p}\left({\langle f\rangle }_{p},K\right)⩾\frac{n}{\left|{\langle f^{K}\rangle }_{p}\right|}V_{p}\left({\langle f^{K}\rangle }_{p},K\right)$$

for $K\in K_{e}^{n}$, with equality if and only if $f\equiv f^{K}$ a.e. in $R^{n}$ up to a translation.

By choosing $K={\langle f\rangle }_{p}$, we get

$$\left|{\langle f^{{\langle f\rangle }_{p}}\rangle }_{p}\right|⩾V_{p}\left({\langle f^{{\langle f\rangle }_{p}}\rangle }_{p},{\langle f\rangle }_{p}\right).$$

By Lemma 3, we have ${\langle f^{{\langle f\rangle }_{p}}\rangle }_{p}$ is homothetic to ${\langle f\rangle }_{p}$.

Thus by Theorem 11, we conclude

$${\langle f\rangle }_{p}\subseteq {\langle f^{{\langle f\rangle }_{p}}\rangle }_{p},$$

with ${\langle f\rangle }_{p}={\langle f^{{\langle f\rangle }_{p}}\rangle }_{p}$ if and only if $f=f^{{\langle f\rangle }_{p}}$ almost everywhere (up to translations).  □

For our purpose, it is easier to deal with these normalized versions of $L^{p}$ Petty projection inequalities.

Theorem 12 **$L^{p}$ projection inequality**

*(See* *[47]* *.) If* 1<p<∞ *and K is a convex body, then*

$$\left|{\tilde{\Pi }}_{p}^{\circ }K\right|⩽m_{p,n}|K|$$

*where*

$$m_{p,n}={\left[\frac{\sqrt{\pi }\Gamma (\frac{p+n}{2})}{2\Gamma (\frac{n}{2}+1)\Gamma (\frac{p+1}{2})}\right]}^{n/p}.$$

*Equality holds if and only if K is an ellipsoid.*

Now we give the proof of Theorem 2.

Proof of Theorem 2 By choosing Φ(x)=|v⋅x| in Theorem 9, we see that

(7) $$\Omega_{p}(f)=c_{n,p}{\left|{\tilde{\Pi }}_{p}^{\circ }\left({\langle f\rangle }_{p}\right)\right|}^{-1/n}$$

where $c_{n,p}$ is a suitably chosen constant depending only on n,p.

By Lemma 3 and Lemma 4, we have ${\langle f^{{\langle f\rangle }_{p}}\rangle }_{p}$ is a dilate of ${\langle f\rangle }_{p}$ and

$${\langle f^{{\langle f\rangle }_{p}}\rangle }_{p}⊇{\langle f\rangle }_{p}.$$

Therefore, by the homogeneity of the projection operator, we get

$${\left|{\tilde{\Pi }}_{p}^{\circ }\left({\langle f\rangle }_{p}\right)\right|}^{-1/n}⩾{\left|{\tilde{\Pi }}_{p}^{\circ }\left({\langle f^{{\langle f\rangle }_{p}}\rangle }_{p}\right)\right|}^{-1/n},$$

with equality if and only if $f=f^{K}$, up to translations, for some $K\in K_{e}^{n}$.

By Lemma 3, we know

$$\left|{\langle f^{{\langle f\rangle }_{p}}\rangle }_{p}\right|=\left|{\langle f^{⋆}\rangle }_{p}\right|.$$

Since ${\langle f^{{\langle f\rangle }_{p}}\rangle }_{p}$ is a dilate of ${\langle f\rangle }_{p}$ and ${\langle f^{⋆}\rangle }_{p}$ is a dilate of $B_{1}$. We conclude from Theorem 12 that

$${\left|{\tilde{\Pi }}_{p}^{\circ }\left({\langle f^{{\langle f\rangle }_{p}}\rangle }_{p}\right)\right|}^{-1/n}⩾{\left|{\tilde{\Pi }}_{p}^{\circ }\left({\langle f^{⋆}\rangle }_{p}\right)\right|}^{-1/n},$$

with equality if and only if ${\langle f\rangle }_{p}$ is an ellipsoid.

Now we have

$$\Omega_{p}(f)=\Omega_{p}\left(f^{⋆}\right),$$

which forces $f=f^{K}$ up to some translations for some $K\in K_{e}^{n}$ and ${\langle f^{K}\rangle }_{p}$ to be an ellipsoid.

Therefore, we have

$$f=f^{E}\;\text{almost everywhere}$$

for some ellipsoid *E* in $R^{n}$, up to translations.  □

## 4 Stability

The study of the stability – an issue of special interest in modern analysis – of aforementioned variation problems is of great interest and still not completely settled, although contributions have been made as far as Sobolev, isoperimetric and isocapacitary type inequalities are concerned [3], [4], [5], [6], [15], [13], [19], [23], [24], [27], [28], [29], [33], [34], [52].

Böröczky has established the following quantitative version of the $L^{p}$ projection inequality:

Theorem 13 *(See* *[6]* *.) For* $K\in K_{0}^{n}$ *with* $\delta =\delta_{BM}(K,B_{1})$ *, we have*

$$\frac{\left|{\tilde{\Pi }}_{p}^{\circ }K\right|}{\left|K\right|}⩽\left(1-\gamma \cdot \delta^{792n+840p}\right)\frac{\left|{\tilde{\Pi }}_{p}^{\circ }B_{1}\right|}{\left|B_{1}\right|},$$

*where* γ>0 *depends on n.*

Let $K\in K_{0}^{n}$ be a convex body such that $a|x|⩽h_{K}(x)⩽b|x|$, where a,b∈(0,∞). We denote the deficit $D_{K}(f)$ of *f* by:

$$D_{K}(f)=\frac{\int_{R^{n}}h_{K}^{p}(\nabla f)}{\int_{R^{n}}h_{K}^{p}(\nabla f^{K})}-1.$$

The Euclidean case of the following theorem is due to Cianchi, Fusco, Esposito and Trombetti [13], the general convex case is due to Esposito and Ronca [19].

Theorem 14 *(See* [13], [19] *.) Let* p>1 *,* n>2 *and* $K\in K_{0}^{n}$ *. There exist positive constants* $r_{1},r_{2},r_{3}$ *and C depending only on p, n, a and b, such that for every* $f\in W^{1,p}(R^{n})$ *satisfying* |({|f>0|})|<∞ *,*

$$\min_{\pm }\underset{x_{0}\in R^{n}}{\inf}\int_{R^{n}}\left|f(x)\pm f^{K}(x+x_{0})\right|\;dx⩽C{\left\|h_{K}(\nabla f)\right\|}_{p}{\left|\left(\left\{|f|>0\right\}\right)\right|}^{1+\frac{1}{n}-\frac{1}{p}}{\left[M_{f}(\sigma )+D_{K}{\left(f\right)}^{r_{1}}+\frac{{\left\|h_{K}(\nabla f)\right\|}_{p}}{\sigma |({\left\{|f|>0\})\right|}^{\frac{1}{p}}}D_{K}{\left(f\right)}^{r_{2}}\right]}^{r_{3}},$$

*for every* σ>0 *.*

The functional Minkowski problem has strong affine invariance properties.

Lemma 5 *(See* *[51]* *.) If* $f\in W^{1,p}(R^{n})$ *and τ be a translation in* $R^{n}$ *, then*

$${\langle f\circ \tau \rangle }_{p}={\langle f\rangle }_{p}.$$

Lemma 6 *(See* *[44]* *.) If* $K,L\in K_{0}^{n}$ *and* ψ∈SL(n) *, then*

$$V_{p}(\psi K,\psi L)=V_{p}(K,L).$$

Lemma 7 *(See* *[51]* *.) If* $f\in W^{1,p}(R^{n})$ *and* ψ∈SL(n) *, then*

$${\langle f\circ \psi^{-1}\rangle }_{p}=\psi {\langle f\rangle }_{p}.$$

Lemma 8 *If* $f\in W^{1,p}(R^{n})$ *and* ψ∈SL(n) *, then*

$$\int_{R^{n}}h_{\psi {\langle f\rangle }_{p}}{\left(\nabla \left(f\circ \psi^{-1}\right)\right)}^{p}\;dx=\int_{R^{n}}h_{{\langle f\rangle }_{p}}{\left(\nabla f\right)}^{p}\;dx.$$

Proof By Theorem 9, Lemma 6, and Theorem 10, we have

$$\int_{R^{n}}h_{{\langle f\rangle }_{p}}{\left(\nabla f\right)}^{p}\;dx=\frac{1}{\left|{\langle f\rangle }_{p}\right|}\int_{S^{n-1}}h_{{\langle f\rangle }_{p}}{\left(u\right)}^{p}\;dS_{p}\left({\langle f\rangle }_{p},u\right)=n.$$

Similarly, we have by recalling Lemma 7 that

$$\int_{R^{n}}h_{\psi {\langle f\rangle }_{p}}{\left(\nabla \left(f\circ \psi^{-1}\right)\right)}^{p}\;dx=n.\;□$$

Lemma 9 **Johnʼs lemma**

*If K is an origin-symmetric convex body in* $R^{n}$ *, then there is an origin-centered ellipsoid E such that the inclusions*

E⊆K⊆nE

*hold.*

Lemma 10 *Given a Sobolev function* $f\in W^{1,p}(R^{n})$ *, let* $\delta_{s}(K,B_{1})$ *denote the normalized symmetric difference between K and* $B_{1}$ *, given by* $\delta_{s}(K,B_{1})=\frac{\left|K△B_{1}\right|}{\left|K\right|}$ *, where* $\left|K|=|B_{1}\right|$ *. Then we have*

$${\left(e^{\delta_{BM}({\langle f\rangle }_{p},B_{1})}\right)}^{n}-1⩾\delta_{s}\left(\psi \tilde{\;{\langle f\rangle }_{p}\;},B_{1}\right),$$

*for some* ψ∈SL(n) *.*

Proof Without loss of generality, assume that

$$\delta_{BM}\left({\langle f\rangle }_{p},B_{1}\right)=R,$$

where $B_{1}\subseteq \psi {\langle f\rangle }_{p}\subseteq e^{R}B_{1}$ for some ψ∈SL(n).

Therefore we have

$$\delta_{s}\left(\psi {\langle f\rangle }_{p},cB_{1}\right)=\frac{\left|\psi {\langle f\rangle }_{p}△cB_{1}\right|}{\left|cB_{1}\right|},$$

where c>1 is chosen such that $\left|cB_{1}|=|{\langle f\rangle }_{p}\right|$.

Since $\psi {\langle f\rangle }_{p}△cB_{1}\subseteq e^{R}B_{1}△B_{1}$, we have

$$\delta_{s}\left(\psi \tilde{\;{\langle f\rangle }_{p}\;},B_{1}\right)=\delta_{s}\left(\psi {\langle f\rangle }_{p},cB_{1}\right)⩽{\left(e^{R}\right)}^{n}-1.\;□$$

We are ready to establish the following quantitative estimate for the affine Pólya–Szegö principle.

Proof of Theorem 3 By recalling Theorem 9, we find that the affine Pólya–Szegö principle could be stated as:

$$c_{n,p}n^{-\frac{1}{n}}{\left(\left|{\tilde{\Pi }}_{p}^{\circ }{\langle f\rangle }_{p}\right|\right)}^{-\frac{1}{n}}⩾c_{n,p}n^{-\frac{1}{n}}{\left(\left|{\tilde{\Pi }}_{p}^{\circ }{\langle f^{{\langle f\rangle }_{p}}\rangle }_{p}\right|\right)}^{-\frac{1}{n}}⩾c_{n,p}n^{-\frac{1}{n}}{\left(\left|{\tilde{\Pi }}_{p}^{\circ }{\langle f^{⋆}\rangle }_{p}\right|\right)}^{-\frac{1}{n}}.$$

Without loss of generality, we assume that the Banach–Mazur distance between ${\langle f\rangle }_{p}$ and *B* is attained by $\ln\;\frac{R}{r}$ where

$$r\psi {\langle f\rangle }_{p}\subset B\subset R\psi {\langle f\rangle }_{p},$$

here 0<r⩽R<∞, ψ∈SL(n) and *B* is an origin-centered ball.

And we assume

$$\min_{\pm }\underset{x_{0}\in R^{n}}{\inf}\int_{R^{n}}\left|f\circ \psi^{-1}(x)\pm f^{\psi {\langle f\rangle }_{p}}(x+x_{0})\right|\;dx=\int_{R^{n}}\left|f\circ \psi^{-1}(x)-f^{\psi {\langle f\rangle }_{p}}(x+\tau_{0})\right|\;dx$$

for convenience.

Since $A_{a}(\cdot )$ is invariant under SL(n) and

$${\langle f\circ \psi^{-1}\rangle }_{p}=\psi {\langle f\rangle }_{p}$$

for ψ∈SL(n), we could assume ${\langle f\rangle }_{p}$ is in a position where there is an origin-centered ball $B^{\prime }$ such that

$$B^{\prime }\subseteq \psi {\langle f\rangle }_{p}\subseteq nB^{\prime }.$$

From the triangle inequality, we have

$$A_{a}(f)⩽{\left\|f\circ \psi^{-1}(x)-f^{\psi {\langle f\rangle }_{p}}(x+\tau_{0})\right\|}_{1}+{\left\|f^{\psi {\langle f\rangle }_{p}}(x+\tau_{0})-f^{B}(x+\tau_{0})\right\|}_{1}.$$

Therefore either

$${\left\|f\circ \psi^{-1}-f^{\psi {\langle f\rangle }_{p}}\right\|}_{1}⩾\frac{1}{2}A_{a}(f)$$

or

$${\left\|f^{\psi {\langle f\rangle }_{p}}-f^{B}\right\|}_{1}⩾\frac{1}{2}A_{a}(f).$$

If ${\left\|f\circ \psi^{-1}-f^{\psi {\langle f\rangle }_{p}}\right\|}_{1}⩾\frac{1}{2}A_{a}(f)$, by Lemma 4, we have

(8) $${\langle f^{{\langle f\rangle }_{p}}\rangle }_{p}=(1+c){\langle f\rangle }_{p},$$

where c⩾0.

We have

$$d|\nabla f|<h_{\tilde{{\langle f\rangle }_{p}}}(\nabla f)<e|\nabla f|,$$

where *d*, *e* are dimensional constants, as $B\subseteq {\langle f\rangle }_{p}\subseteq nB$ for some origin-centered ball *B*.

Notice

$$R_{a}(f)⩾\frac{{\left|{\tilde{\Pi }}_{p}^{\circ }{\langle f\rangle }_{p}\right|}^{-1/n}}{{\left|{\tilde{\Pi }}_{p}^{\circ }{\langle f^{{\langle f\rangle }_{p}}\rangle }_{p}\right|}^{-1/n}}=1+c.$$

By the definition of ${\langle f\rangle }_{p}$, we have

$$\int_{R^{n}}h_{\tilde{{\langle f\rangle }_{p}}}{\left(\nabla f\right)}^{p}\;dx=\frac{1}{\left|{\langle f\rangle }_{p}\right|}\int_{S^{n-1}}h_{\tilde{{\langle f\rangle }_{p}}}{\left(u\right)}^{p}\;dS_{p}\left({\langle f\rangle }_{p},u\right)$$

and

$$\int_{R^{n}}h_{\tilde{{\langle f\rangle }_{p}}}{\left(\nabla f^{{\langle f\rangle }_{p}}\right)}^{p}\;dx=\frac{1}{\left|{\langle f^{{\langle f\rangle }_{p}}\rangle }_{p}\right|}\int_{S^{n-1}}h_{\tilde{{\langle f\rangle }_{p}}}{\left(u\right)}^{p}\;dS_{p}\left({\langle f^{{\langle f\rangle }_{p}}\rangle }_{p},u\right).$$

Thus, using (8), we observe

$$\frac{\int_{R^{n}}h_{\tilde{{\langle f\rangle }_{p}}}{\left(\nabla f\right)}^{p}\;dx}{\int_{R^{n}}h_{\tilde{{\langle f\rangle }_{p}}}{\left(\nabla f^{{\langle f\rangle }_{p}}\right)}^{p}\;dx}-1={\left(1+c\right)}^{p}-1⩽{\left(R_{a}(f)\right)}^{p}-1.$$

Therefore, by Theorem 14, Lemma 8 and the Hölder Inequality, we have

$$\underset{x_{0}\in R^{n}}{\inf}\int_{R^{n}}\left|f\circ \psi^{-1}(x)-f^{\psi {\langle f\rangle }_{p}}(x+x_{0})\right|\;dx⩽C{\left\|f\right\|}_{W^{1,p}(R^{n})}{\left|\left(\left\{|f|>0\right\}\right)\right|}^{1+\frac{1}{n}-\frac{1}{p}}{\left[M_{f}(\sigma )+D_{{\langle f\rangle }_{p}}{\left(f\right)}^{r_{1}}+\frac{{\left\|f\right\|}_{W^{1,p}(R^{n})}}{\sigma |({\left\{|f|>0\})\right|}^{\frac{1}{p}}}D_{{\langle f\rangle }_{p}}{\left(f\right)}^{r_{2}}\right]}^{r_{3}}⩽C{\left\|f\right\|}_{W^{1,p}(R^{n})}{\left|\left(\left\{|f|>0\right\}\right)\right\|}^{1+\frac{1}{n}-\frac{1}{p}}{\left[M_{f}(\sigma )+{\left({\left(R_{a}(f)\right)}^{p}-1\right)}^{r_{1}}+\frac{{\left\|f\right\|}_{W^{1,p}(R^{n})}}{\sigma |({\left\{|f|>0\})\right|}^{\frac{1}{p}}}{\left({\left(R_{a}(f)\right)}^{p}-1\right)}^{r_{2}}\right]}^{r_{3}}$$

for every σ>0, here $C,r_{1},r_{2},r_{3}$ are constants depending only on *n* and *p*.

On the other hand, using the layer-cake representation and the Fubini theorem, we have

$${\left\|f^{\psi {\langle f\rangle }_{p}}-f^{B}\right\|}_{1}=\int_{R^{n}}\left|\int_{0}^{\infty }\chi_{\left\{f^{\psi {\langle f\rangle }_{p}}>t\right\}}(x)\;dt-\int_{0}^{\infty }\chi_{\left\{f^{B}>t\right\}}(x)\;dt\right|\;dx⩽\int_{R^{n}}\int_{0}^{\infty }\left|\chi_{\left\{f^{\psi {\langle f\rangle }_{p}}>t\right\}}(x)-\chi_{\left\{f^{B}>t\right\}}(x)\right|\;dt\;dx=\int_{0}^{\infty }|E_{t}|\;dt,$$

where $E_{t}=\{{\left[f^{\psi {\langle f\rangle }_{p}}\right]}_{t}△{\left[f^{B}\right]}_{t}\}$.

Since

$$\left|{\left[f^{\psi {\langle f\rangle }_{p}}\right]}_{t}\right|=\left|{\left[f^{B}\right]}_{t}\right|,$$

we have

$${\left\|f^{\psi {\langle f\rangle }_{p}}-f^{B}\right\|}_{1}=\int_{0}^{\infty }\delta_{s}\left({\left[f^{\psi {\langle f\rangle }_{p}}\right]}_{t},{\left[f^{B}\right]}_{t}\right)\left|{\left[f^{B}\right]}_{t}\right|\;dt.$$

Since

$$\delta_{s}\left({\left[f^{\psi {\langle f\rangle }_{p}}\right]}_{t},{\left[f^{B}\right]}_{t}\right)$$

is a constant for t>0 a.e., we have

$${\left\|f^{\psi {\langle f\rangle }_{p}}-f^{B}\right\|}_{1}⩽\delta_{s}\left({\left[f^{\psi {\langle f\rangle }_{p}}\right]}_{t},{\left[f^{B}\right]}_{t}\right){\left\|f\right\|}_{L^{1}(R^{n})}⩽\delta_{s}\left(\tilde{\;{\langle f\rangle }_{p}\;},B_{1}\right){\left\|f\right\|}_{L^{1}(R^{n})}.$$

Notice that, from Lemma 3, we have

$$\left|{\langle f^{{\langle f\rangle }_{p}}\rangle }_{p}\right|=\left|{\langle f^{B}\rangle }_{p}\right|.$$

And

$$\Omega_{p}(f)=c_{n,p}n^{-\frac{1}{n}}{\left(\left|{\tilde{\Pi }}_{p}^{\circ }{\langle f\rangle }_{p}\right|\right)}^{-\frac{1}{n}},$$

thus

$${\left(R_{a}(f)\right)}^{-n}⩽\frac{\left|{\tilde{\Pi }}_{p}^{\circ }\tilde{\;{\langle f\rangle }_{p}\;}\right|}{\left|{\tilde{\Pi }}_{p}^{\circ }B_{1}\right|}⩽1-\gamma_{n}\cdot \delta_{BM}{\left(\tilde{\;{\langle f\rangle }_{p}\;},B_{1}\right)}^{792n+840p}.$$

Thus we have

$$\delta_{BM}\left(\tilde{\;{\langle f\rangle }_{p}\;},B_{1}\right)⩽{\left(\frac{1-{\left(R_{a}(f)\right)}^{-n}}{\gamma_{n}}\right)}^{\frac{1}{792n+840p}}.$$

From Lemma 10, we have

$$\delta_{s}\left(\tilde{\;{\langle f\rangle }_{p}\;},B\right)⩽e^{{\left(\frac{1-{\left(R_{a}(f)\right)}^{-n}}{\gamma_{n}}\right)}^{\frac{n}{792n+840p}}}-1.$$

Thus we have

$${\left\|f^{\psi {\langle f\rangle }_{p}}-f^{B}\right\|}_{1}⩽\left(e^{{\left(\frac{1-{\left(R_{a}(f)\right)}^{-n}}{\gamma_{n}}\right)}^{\frac{n}{792n+840p}}}-1\right){\left\|f\right\|}_{1}⩽\left(e^{{\left(\frac{1-{\left(R_{a}(f)\right)}^{-n}}{\gamma_{n}}\right)}^{\frac{n}{792n+840p}}}-1\right){\left|\left\{|f|>0\right\}\right|}^{\frac{p-1}{p}}{\left\|f\right\|}_{W^{1,p}(R^{n})}.$$

Thus, by triangle inequality, we have

$$A_{a}(f)⩽C{\left\|f\right\|}_{W^{1,p}(R^{n})}{\left|\left(\left\{|f|>0\right\}\right)\right|}^{1+\frac{1}{n}-\frac{1}{p}}{\left[M_{f}(\sigma )+{\left({\left(R_{a}(f)\right)}^{p}-1\right)}^{r_{1}}+\frac{{\left\|f\right\|}_{W^{1,p}(R^{n})}}{\sigma |({\left\{|f|>0\})\right|}^{\frac{1}{p}}}{\left({\left(R_{a}(f)\right)}^{p}-1\right)}^{r_{2}}\right]}^{r_{3}}+2\left(e^{{\left(\frac{1-{\left(R_{a}(f)\right)}^{-n}}{\gamma_{n}}\right)}^{\frac{n}{792n+840p}}}-1\right){\left|\left\{|f|>0\right\}\right|}^{\frac{p-1}{p}}{\left\|f\right\|}_{W^{1,p}(R^{n})}.$$

The theorem is proved once we recall that |{x:∇f(x)≠0}|=1 and ${\left\|f\right\|}_{W^{1,p}(R^{n})}=1$.  □

Corollary 1 *Let f be a function from* $W^{1,p}(R^{n}),1<p<+\infty$ *, such that* $|\{|Df|=0\}\cap {\left(f\right)}^{-1}(0,\mathrm{ess}\sup|f|)|=0$ *, and* |{|f|>0}|<∞ *. If*

$$\Omega_{p}\left(f^{⋆}\right)=\Omega_{p}(f),$$

*then either*

$$f=f^{⋆}\circ \psi$$

*or*

$$f=-f^{⋆}\circ \psi$$

*for some* ψ∈SL(n) *a.e. up to a translation.*

Proof By taking $R_{a}(f)=1$ and choosing σ→0 in the proof of Theorem 3, we are done.  □
